# What to expect for the influenza season 2020/21 with the ongoing COVID-19 pandemic in the World Health Organization European Region

**DOI:** 10.2807/1560-7917.ES.2020.25.42.2001816

**Published:** 2020-10-22

**Authors:** Cornelia Adlhoch, Richard Pebody

**Affiliations:** 1 European Centre for Disease Prevention and Control, Stockholm, Sweden; 2 World Health Organization Regional Office for Europe, Copenhagen, Denmark

**Keywords:** influenza, surveillance, WHO European Region, COVID-19

One constant feature of the northern hemisphere’s winter months is the circulation of influenza viruses, leading to seasonal epidemics. In years when a novel influenza virus appears, such as in 2009, this can lead to a pandemic.

In 2020, the emergence of coronavirus disease (COVID-19) caused by the severe acute respiratory syndrome coronavirus 2 (SARS-CoV-2) has affected people all across the World Health Organization (WHO) European Region and beyond, forcing everyone to rapidly adapt and change personal and professional ways of life. Based on observations to date, the forthcoming 2020/21 influenza season is also likely to be different from what we might usually expect.

In response to increasing circulation of SARS-CoV-2 in Europe in spring 2020, many countries implemented a range of strict non-pharmaceutical public health measures to reduce virus spread. These included stay-at-home orders, movement restrictions, face mask use and school closures. In addition to a subsequent reduction in COVID-19 incidence, these interventions were associated with an apparent and rapid decrease in infections with other respiratory viruses such as influenza and respiratory syncytial virus (RSV) [1]. Though questions were raised whether this observation reflected a true decrease in transmission or rather a disruption of existing surveillance systems, triangulating the evidence suggests the former explanation is more likely. Firstly, the 2019/20 influenza season had a shorter period of high influenza activity with a positivity rate > 50% (2 weeks vs. 5–8 weeks; Figure) and ended earlier than usual, with the positivity rate of sentinel specimens decreasing more rapidly to < 10% (18 weeks vs. 19–25 weeks for total length of season) compared with the previous five seasons (2014/15–2018/19) [1,2]. Secondly, syndromic data on patient consultation rates for influenza-like illness (ILI) or acute respiratory symptoms (ARI) in primary care decreased to very low levels by weeks 13–14 (in mid-March), where they remained over the summer period across the European Region. This was likely due to lower respiratory infections in the population, restricted access to primary health care and reduced reporting of data [3]. Thirdly, although the number of influenza-tested sentinel specimens remained at comparable levels with previous inter-seasonal periods (weeks 21–39), only three of the 1,532 tested sentinel specimens were positive, for influenza B/no lineage, B/Victoria and A(H3) virus, respectively. The positivity rate of 0.2% in the 2020 inter-seasonal period was lower than the average (1.1%) observed over the previous five inter-seasonal periods. Moreover, although the number of tested specimens in non-sentinel surveillance systems increased by almost 50% (72,132 vs 51,403 specimens) compared with the average over the previous five inter-seasonal periods, only 46 specimens were positive for influenza (A unsubtyped n = 19; A(H1)pdm09 n = 3; A(H3) n = 6; B/no lineage n = 18; Table). These findings confirm that these systems—which obtain specimens mainly from hospital settings, outbreak investigations and primary care diagnostic laboratories—continued to carry out widespread testing for influenza during the summer period. Despite this extensive testing, very few influenza viruses were detected and the resulting positivity rate of 0.1% was much lower than the historical average of 1.8% (Table).

**Table:** Number and percentage of tested and influenza-positive specimens in sentinel and non-sentinel surveillance, World Health Organization European Region, inter-seasonal weeks 21–39, 2014–2020

Year	Sentinel			Non-sentinel		
	Detections		Specimens tested	Detections		Specimens tested
	N	%		N	%	
2014	9	1.7%	522	329	1.3%	24,849
2015	20	1.1%	1,902	949	1.9%	50,462
2016	29	1.4%	2,083	1,005	1.8%	55,292
2017	37	1.3%	2,904	1,422	2.6%	55,557
2018	10	0.4%	2,399	568	1.1%	52,138
2019	9	0.6%	1,620	1,310	1.9%	70,122
2020	3	0.2%	1,532	46	0.1%	72,132

Source: The European Surveillance System (TESSy); data as at week 41/2020.

Similar inter-seasonal findings were reported from the United States and from the temperate southern hemisphere during its 2020 winter season [4]. Indeed, data from Australia, Chile and South Africa for the period April–July 2020 show that only 51 influenza virus detections were made from 83,307 tested samples (0.06%), compared with 24,512 from 178,690 tested (13.7%) over the same period in the years 2017–2019. Community mitigation measures were seen as the most likely explanation for this observation [5-7], though other potential explanations such as viral interference were raised as well [8].

Although global influenza circulation remains at lower levels than expected as at early autumn [4,9,10], sporadic detection of influenza viruses during the northern hemisphere’s summer reminds us that circulation is ongoing, particularly in parts of the tropics and sub-tropics where influenza circulates year-round without clear seasonality. There have been reports of severe cases admitted to intensive care units because of influenza A(H1N1)pdm09 and at least one fatal outcome during the summer months [11]. Recently, the emergence of A(H1N1)pdm09 virus antigenic variants in the genetic group 5A-156K was reported, leading to a change in the recommended vaccine composition for the 2021 southern hemisphere influenza season [12]. The potential for global spread, which is recognised as linked to international air travel, remains valid [13]. All of these factors highlight the importance of ongoing vigilance and sustained influenza surveillance.

Following a period of reduced COVID-19 transmission over the summer, most countries across the European Region are now seeing an upsurge in COVID-19 cases [3] and are again implementing various public health and social measures. The continuation or re-implementation of several of these public health and social measures will likely lead to an overall reduction in circulating influenza viruses during the upcoming winter months compared with previous seasons, particularly considering the lower basic reproduction number (R0) of influenza compared with SARS-CoV-2 [14]. However, the re-opening of schools in most countries, which is recognised as a key driver for influenza transmission, increases the risk of influenza virus circulation. While the start of the northern hemisphere seasonal epidemic is usually around the end of November or early December [2,15], it is important to note that a shift of timing, e.g. later than usual, in the forthcoming influenza season could be one possible scenario.

In many countries, the response to COVID-19 may include a redirection of primary care patients to COVID-19 centres, reducing access to primary care sentinel surveillance sites. Thus, prioritising COVID-19 testing has the potential to impact established influenza surveillance during the winter months. To mitigate such consequences, joint European Centre for Disease Prevention and Control (ECDC) and WHO Regional Office for Europe (WHO/Europe) influenza surveillance interim guidance for the upcoming winter season has been published to help countries better perform sustained and integrated sentinel surveillance [8]. This is required for several reasons. Sentinel surveillance systems are designed to be representative of the outpatient population and can serve as a good indicator of early community activity of influenza, but also other respiratory illnesses such as COVID-19, throughout the European Region as well as locally and nationally. Virus characterisation data are needed to guide global decision-making for influenza vaccine composition for the northern hemisphere’s 2021/22 season and beyond, but also for monitoring the evolution of SARS-CoV-2. Additionally, detailed virus characterisation analyses of specimens of unsubtypable influenza viruses from routine surveillance systems will help to identify zoonotic transmission events, such as the described case of a swine influenza virus A/sw/H1avN1 in a child in this issue [16]. Finally, co-infections with influenza virus and SARS-CoV-2 might be associated with higher severity and need to be further investigated [17]. The interim guidance recommends that case definitions for ILI and/or ARI in primary care sentinel sites and severe acute respiratory infection (SARI) in secondary care should continue to be applied to allow historical comparability. Sampled patients should be tested concurrently for influenza virus and SARS-CoV-2, ideally with multiplex assays, if available, to save resources.

The notion that influenza is unpredictable remains true for the 2020/21 season. There are many unknowns regarding the timing and intensity of influenza activity this winter, as well as what will be the dominant circulating virus type, subtype or lineage. Seasonal influenza vaccination efforts remain important to minimise influenza-related disease burden. Temporary recommendations have been issued for this season to prioritise healthcare workers, elderly people and other risk groups in an effort to safeguard the healthcare system by reducing the impact of influenza [18]. Guidance on how these priority groups can be vaccinated safely during the ongoing pandemic has also been issued.

In summary, although COVID-19 prevention and control measures will also support influenza prevention, influenza remains a threat to human health and a potential burden on the healthcare system. Continued monitoring and vaccination, particularly during the ongoing COVID-19 pandemic, is therefore required. Surveillance and healthcare systems need to remain flexible and adapt to the challenges ahead. We highlight the joint ECDC and WHO/Europe guidance to maintain influenza surveillance systems both in primary and secondary care this winter [8]. This will ensure virological and epidemiological data are available for situational assessment of the intensity and impact of influenza viruses and will also inform the selection of vaccine components for the February 2021 vaccine composition meeting.
